# Editorial: Nutrition and oral health: Public health relevance

**DOI:** 10.3389/froh.2023.1130786

**Published:** 2023-01-26

**Authors:** Mainul Haque, Md. Anwarul Azim Majumder, Mohammed S. Razzaque

**Affiliations:** ^1^ National Defense University of Malaysia, Kuala Lumpur, Malaysia; ^2^ The University of the West Indies, Bridgetown, Barbados; ^3^ Lake Erie College of Osteopathic Medicine, Erie, PA, United States

**Keywords:** oral - general health, nutrition, public health, oral microbiota, tea

Editorial on the Research Topic Nutrition and oral health: Public health relevance

The roles and regulation of various nutrients in systemic health are well known, although the bifacial association among oral health, diet, and nutrition are not studied in similar depth and detail. Nutritional imbalances, including vitamins C and D, magnesium, zinc, calcium, and phosphate, are shown to be associated with the initiation and propagation of various chronic oral diseases (1–5), including gingivitis and dental decay (6, 7). Sugar-sweetened beverages are also linked to a higher risk of developing non-communicable diseases and oral diseases in low-, middle- and high-income countries (8, 9). Oral manifestations are one of the earliest clues of the evolvement of some prevalent systemic diseases, including metabolic disorders (1, 10). This Research Topic is intended to bring together different dental, medical, and nutritional science specialties to share their experiences in reducing oral health burden and improving systemic diseases and beyond; 17 articles were published on this Research Topic to attain the intended goals.

The oral microbiota is vital to the human microbiome (11). Native microbiota of the oral cavity could prevent adhesion and invasion of pathogens on the oral mucosa to facilitate colonization resistance. The balance between bacterial symbiosis, microbial virulence, and host resistance ensures the integrity of the oral cavity. Akimbekov et al. explained how nutritional factors impact the integrity of the oral indigenous microbiota and its contribution to colonization resistance.

Orthodontic patients are at significant risk for developing oral lesions by facilitating increased plaque formation and bacterial dysbiosis in the oral cavity. Oral probiotics with efficacy against caries offer an attractive option to reduce caries risk in these patients. In a randomized controlled trial, Ebrahim et al. determined the effectiveness of a commercially obtainable Lorodent Probiotic Complex at reducing plaque buildup and Streptococcus mutans bacterial accumulations in adolescent orthodontic patients. Although no significant changes in the oral outcome measures were found in the study, the results offer a baseline for subsequent testing of other potential probiotics in adolescents. Koukou et al. reviewed the possible association between fixed orthodontic treatment and the onset of eating disorders. While limited numbers of case reports suggest that patients develop eating disorders after the initiation of fixed orthodontic treatment, well-designed clinical studies are needed to determine whether fixed orthodontic treatment is the cause of eating disorders.

Several articles on this Research Topic focused on the roles and regulations of vitamins, minerals, and sugar-sweetened beverages in the initiation and progression of various oral diseases. In a population-based study in Brazil, Nascimento et al. found a protective effect of calcium, but not vitamin D, on periodontitis, mainly among women. In another Brazilian study, Feldens et al. provided recommendations to improve the oral health of the populations. The Brazilian Academy of Dentistry recommended no sugar-containing food to less than 2 years of old children and limiting total sugar consumption to <25 gm/day after 2 years of age. In a systematic review, Zupo et al. identified that alcohol, sugary drinks, and coffee consumption were associated with poor oral health outcomes in the elderly population, including periodontal disease, oral dysbiosis, and tooth loss.

Santosh et al. highlighted the global health burden, economic impact, and oral health inequalities, focusing on the Caribbean region. Mahriani et al. reported the relationship between oral hygiene behavior and adolescent girls’ oral health status. The authors found that the higher the oral hygiene behavior score, the higher the oral health condition score, which could be seen in association with the family's socioeconomic status. Al Anouti et al. summarized the accessible data on oral health among children and adolescents in the United Arab Emirates (UAE) over the past decade for developing future strategies to effectively implement preventive and interventional programs.

Dai et al. studied the relationship between serum 25-hydroxyvitamin D concentration and all-cause and cause-specific mortality among adult patients with existing cardiovascular disease. In a large prospective cohort study with 37,080 cardiovascular disease patients, the increase in serum 25(OH)D levels were found to be associated with a reduced risk of all-cause and cause-specific mortality, and the decreasing trend of mortality risk reached a plateau at around 50 nmol/L concentration of 25-hydroxyvitamin D. Huang et al. using the National Health and Nutrition Examination Survey (NHANES) database, reported that blood lead and cadmium levels were positively associated with mean clinical attachment loss with periodontitis, while blood selenium was negatively associated with mean clinical attachment loss. Further research is warranted to determine the underlying mechanism of trace minerals dysregulation. Using the China Health and Nutrition Survey (2000–2011), Qi et al. reported that systolic-diastolic hypertension increased with higher carbohydrate energy intake, which was not observed in isolated systolic hypertension, nor in isolated diastolic hypertension in men and women. A cross-sectional study was performed by Majdi et al. to determine the association between habitual and meal-specific carbohydrate quality index and metabolic syndrome among Iranian adults, but no such association was documented. However, the quantity and quality of the food, eating time, and frequency of eating should be considered for developing healthy eating habits. Murererehe et al. elaborated on the protective roles of vitamin C in reducing oral disease burdens, ranging from cariogenesis to carcinogenesis.

Fluorosis is caused by excessive fluoride intake through drinking water, using fluoride supplementation, or using fluoridated toothpaste. In addition, excessive consumption of brick tea in Tibetan areas can also induce fluorosis. The article by Wen et al. reported the prevalence of dental fluorosis and its relationship with brick-tea consumption among the Tibetan residential area population. Of clinical importance, maternal consumption of fluorinated brick tea may be associated with dental fluorosis in children. A higher probability of brick-tea fluorosis is associated with a higher altitude, and based on the results, Wen et al. recommended the restriction of excessive consumption of high-fluoride brick-tea to avoid dental fluorosis. Deng et al. assessed the potential effect of oolong tea consumption on the risk of oral squamous cell cancer, using 744 newly diagnosed oral squamous cell cancer patients and 1,029 healthy controls. Compared to their non-drink counterparts, patients who drank oolong tea demonstrated a reduced risk of oral squamous cell cancer. Moreover, the reduced risk was associated with tea-drinking habits (for detail, please see the article). Furthermore, subgroup analysis revealed that poor oral hygiene was a confounding factor in the negative association of oolong tea drinking with oral squamous cell cancer risk. Aslam et al. used a novel mathematical model of trimmed regression to determine the relationship between dietary fat consumption and prostate cancer. The authors claimed that the proposed model effectively forecasts prostate cancer patients under an indeterminacy setting.

The Global Burden of Disease Study 2017 has estimated nearly 3.5 billion people around the globe are affected by oral infections and other diseases (12). In low- and middle-income countries with rapid urbanization, lifestyle alterations and dietary behaviors are partly contributed to a higher prevalence of oral diseases. Such a higher rate of oral diseases is predominantly due to nutritional inadequacy and lack of access to primary oral health care.

In summary, the aforementioned articles published in this Research Topic highlight the dietary aspects of closely connected oral lesions, such as the harmful effects of brick-tea in dental fluorosis and the potentially beneficial effects of oolong tea in oral tumorigenesis. These articles also help increase the healthcare providers’ awareness of the importance of maintaining nutritional balance to prevent or delay the emergence of oral diseases. Together, this Research Topic highlighted the need for discussion within the healthcare providing community to develop effective nutritional strategies, intended to promote healthier food consumption habits by reducing disease burden and improving oral health for all age range.

## Author contributions

MR wrote the first draft. MH, AM and MR revised and approved the final submitted version.
